# Rationale and design of the costs, health status and outcomes in community-acquired pneumonia (CHO-CAP) study in elderly persons hospitalized with CAP

**DOI:** 10.1186/1471-2334-13-597

**Published:** 2013-12-19

**Authors:** Marie-Josée J Mangen, Marc JM Bonten, G Ardine de Wit

**Affiliations:** 1 Julius Center for Health Sciences and Primary Care, University Medical Center Utrecht, Heidelberglaan 100, Utrecht 3584, CX, The Netherlands; 2 Department of Medical Microbiology, University Medical Center Utrecht, Heidelberglaan 100, Utrecht 3584, CX, The Netherlands; 3 National Institute of Public Health and the Environment, Antonie van Leeuwenhoeklaan 9, Bilthoven 3721, MA, The Netherlands

**Keywords:** Community-acquired pneumonia, Quality-of-life, Healthcare resources use, Non-healthcare resources use, Societal costs

## Abstract

**Background:**

Vaccine effectiveness is usually determined in randomized controlled trials (RCT) and if effective, additional information, e.g. on cost-effectiveness, is required to allow evidence-based decision making. A prerequisite for proper health economic modelling is the availability of good quality data on health care resources use, health outcomes and quality-of-life (QoL) data. The “Collecting health outcomes and economic data on hospitalized Community Acquired Pneumonia (CHO-CAP) – a prospective cohort study” is executed alongside the Community Acquired Pneumonia Immunization Trial with Adults (CAPiTA trial) to capture health outcomes and economic data of elderly hospitalized with CAP and matched controls without CAP.

**Methods/Design:**

CAPiTA is a placebo-controlled double-blind RCT evaluating the effectiveness of a 13-valent conjugated pneumococcal vaccine in preventing vaccine-type pneumococcal CAP in 84,496 elderly in the Netherlands. Participants of CAPiTA, who consented and provided information on health status (EQ-5D) and socio-demographic background at the time of vaccination, constitute the source population of CHO-CAP and are eligible for the nested matched cohort study. CHO-CAP patients hospitalized with CAP form the “diseased” cohort and the “non-diseased” cohort consists of unaffected persons (i.e. no CAP). Observations in the diseased cohort and in matched controls from the non-diseased cohort are used to determine excess costs and QoL changes attributable to CAP.

Based on an estimated 2,000 CAPiTA participants being hospitalized with CAP and an assumed CHO-CAP participation rate of 30% of all CAPiTA participants (±25,000), 600 CAP episodes are expected among CHO-CAP participants (the “diseased” cohort). For each patient with CAP, two non-diseased CHO-CAP subjects will be selected from the CHO-CAP cohort, with matching for age, gender and EQ-5D baseline-score. Data on healthcare and non-healthcare resources use, quality-of-life (using EQ-5D and SF-36 questionnaires) and selected health outcomes will be collected at 0, 1, 6 and 12 months after hospitalization for CAP.

The CHO-CAP study was approved by the Central Committee on Research involving Human Subjects in the Netherlands.

**Discussion:**

With an expected 600 CAP episodes this study will be one of the biggest prospectively studied cohorts of hospitalized elderly with CAP with regard to resources use and Qol data. Strengths of this study further include collection of out-of-pocket costs of patients and productivity losses of both patients and their caregivers and the follow-up period of up to one year post-discharge. This study is therefore expected to add more in-depth knowledge on the short and longer term outcomes of pneumonia in elderly.

**Trial registration:**

ClinicalTrials.gov, NCT00812084.

## Background

The Community Acquired Pneumonia Immunization Trial in Adults (CAPiTA) is a randomized placebo-controlled double-blind trial to determine the efficacy of a 13-Valent Pneumococcal Conjugate Vaccine (13vPnC) in the prevention of vaccine-serotype pneumococcal community-acquired pneumonia (CAP) and invasive pneumococcal disease (IPD) [1]. Within the CAPiTA trial, 84,496 volunteers aged 65 and over, dispersed over the Netherlands, received 13vPnC or placebo in a single-dose vaccine between October 2008 and 31 January 2010. Objectives with regard to efficacy, safety and health outcomes have been described elsewhere [1]. In case of clinical vaccine effectiveness for CAP prevention, drug-licensing and governmental bodies will require detailed information on the cost-effectiveness of this vaccine in elderly in order to decide whether restricted (financial) resources have to be invested in widespread use of this vaccine [2,3]. If clinically effective, this vaccine might reduce healthcare resource use, increase quality-of-life and reduce mortality in elderly. These effects stretch beyond the hospitalization period, as covered in the CAPiTA trial.

There are few recent cost estimates for CAP, and none of them have been derived from Dutch patients [4]. The few prospectively conducted studies (e.g. [5-8]) considered only direct healthcare costs during hospitalisation, had no or limited follow-up post-discharge, and most had a relative small sample size [7,8]. Larger cost studies were mainly retrospective, focussing mostly on direct healthcare costs of patients hospitalised with CAP, with no or limited follow-up post-discharge [4,7,9-18]. However, since CAP may influence the occurrence of other disease events (i.e. stroke and other cardiovascular events [19,20]) in the post-discharge period, it is important to include all healthcare resource use, not only resource use related to the index CAP episode. Also, post-discharge mortality is non-negligible in this population [21]. It is therefore important to have a follow-up period that is much longer than the immediate CAP-related hospitalization period when estimating costs associated with CAP.

The “Costs, Health status and Outcomes of CAP” study (CHO-CAP), in full “Collecting health outcomes and economic data on hospitalized Community-Acquired Pneumonia – a prospective cohort study”, is designed to prospectively determine health outcomes, quality-of-life (QoL) and costs associated with CAP for a period of 12 months after hospitalization due to CAP.

### Objective

CHO-CAP comprises the collection of cost data and quality-of-life data in elderly with and without CAP. The primary objectives are:

1. To determine, during a 12 month follow-up period, differences in quality-of-life of elderly with and without CAP.

2. To determine, during a 12 month follow-up period, differences in resource use (healthcare and non-healthcare) in elderly with and without CAP.

The secondary objective is to describe the baseline health status and quality-of-life of a community-dwelling elderly population eligible for participation in the CAPiTA study.

## Methods/Design

### Ethical and governance approval

Approval was granted by the Central Committee on Research involving Human Subjects (in Dutch Centrale Commissie Mensgebonden Onderzoek (CCMO)), Ref: NL.24770.041.08.

### Study design

CHO-CAP consists of a baseline cohort and a nested matched-cohort study, both executed in parallel to the CAPiTA trial.

#### Baseline cohort

**Figure 1:**
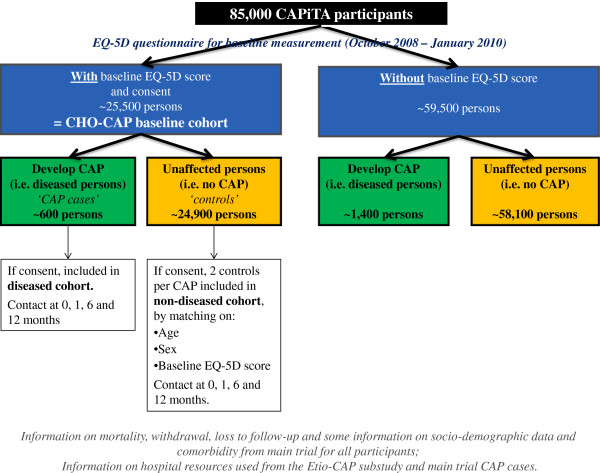
Flow chart of the CHO-CAP study with different (sub-)cohorts and anticipated number of participants.

The study population included in the CAPiTA trial consists of 84,496 community-dwelling persons 65 years and older randomized to receive 13vPnC or placebo (for details of inclusion and exclusion criteria see Hak et al. [1]). All patients included were eligible to participate in the CHO-CAP study. For the baseline cohort, CAPiTA participants were approached at the vaccination centre for participation in CHO-CAP. After inclusion in the CAPiTA study and receipt of study vaccination, participants received written information on the CHO-CAP study. Subjects were asked to fill in a questionnaire and to return it, together with a signed informed consent form, in a pre-stamped envelope. Those who did form the source population (i.e. baseline CHO-CAP cohort) are eligible for participation in the nested matched cohort study (see Figure 1). Subjects returning their questionnaire without informed consent form were contacted a second time with the request to also return a signed informed consent.

#### Nested matched cohort study

As part of the CAPiTA trial, patients with a clinical suspicion of CAP are tracked in 56 Dutch sentinel hospitals. The duration of follow-up within CAPiTA is defined by the number of primary endpoints captured (i.e. 130 first CAP episodes caused by vaccine-type *S. pneumoniae* serotypes). It is expected that around 2,000 CAP episodes will occur within the study population [1]. Assuming a response rate of ~30%^a^ for the baseline questionnaire, we expected approximately 600 first CAP episodes among CHO-CAP participants. Local research nurses in sentinel hospitals notify the CHO-CAP team of newly admitted CAPiTA patients with a clinical suspicion of CAP directly after hospital admission and at the time of discharge. After discharge, eligible patients (i.e. CHO-CAP participants) will be asked to participate in the prospective cohort study for a period of one year. Patients are informed (orally and by written information) that participation involves one interview during a home visit, with three subsequent questionnaires to be completed during the 12 month period. Patients again provide informed consent upon inclusion in the prospective cohort study. Exclusion criteria for the prospective cohort study are hospitalization for a second (or further) hospitalization for CAP related complaints, inability to complete questionnaires, or a recent diagnosis of malignancy as the study was considered too burdensome for this latter group.

For each patient with CAP in the prospective cohort study (“diseased” cohort), two matched unaffected subjects (i.e. no CAP episode until the time of matching) will be selected from the baseline population for inclusion in the “non-diseased” cohort (see Figure 1). Matching is based on age (same age, with allowance of 3 years age difference), gender and baseline health status (same EQ-5D score [22], with maximum allowance of 5 percent points difference). Expectations are that both in the short-term and the medium term, CAP patients consume more medical resources, require more (in)formal help, might experience more trouble to get back to their daily activities, and have a lower quality-of-life than their non-diseased peers. Furthermore, mortality, both within-hospital as during the 12 months follow-up period, is expected to be higher in CAP patients than in controls.

The inclusion of two unaffected controls increases the validity of study results and diminishes the risks from loss to follow-up. Exclusion criteria for controls are admission to hospital for a CAP episode since vaccination, inability to complete questionnaires, or having a recently diagnosed malignancy, as the study was considered too burdensome for the latter group. Controls also provide informed consent at start of follow-up.

### Data collection

#### Baseline CHO-CAP cohort study at time of vaccination

At the time of vaccination subjects were asked to provide information on:

•health status (5 item EQ-5D instrument [22] (see Table 1)),

•socio-demographic status (i.e. education and current living situation)

•self-reporting of previous manifestations of stroke or other cardiovascular disease events.

**Table 1:** **The 5-item EQ-5D questionnaire (Source:**[22]**)**

**Mobility**	
I have no problems in walking about	□
I have some problems in walking about	□
I am confined to bed	□
**Self-care**	
I have no problems with self-care	□
I have some problems washing or dressing myself	□
I am unable to wash or dress myself	□
**Usual activities (e.g. work, study, housework, family or leisure activities)**	
I have no problems with performing my usual activities	□
I have some problems with performing my usual activities	□
I am unable to perform my usual activities	□
**Pain/discomfort**	
I have no pain or discomfort	□
I have moderate pain or discomfort	□
I have extreme pain or discomfort	□
**Anxiety/depression**	
I am not anxious or depressed	□
I am moderately anxious or depressed	□
I am extremely anxious or depressed	□

After receipt of the completed baseline questionnaire, CAPiTA participant ID, informed consent, socio-demographic data, and EQ-5D index value for health status (see below) were registered in the CHO-CAP database, with linkage to the CAPiTA database for age, gender and place of residence/region. Regular updates to adjust for changes in place of residence or to include loss-to-follow up (e.g., due to death), take place.

Following the standards set up by the Euroqol organisation, the developers and owners of the EQ-5D instrument [22], the 5-items from the EQ-5D questionnaire were transferred into a single value between 0 (worst imaginable health status) and 1 (best imaginable health status) [23].

#### Nested matched cohort study

Each identified CHO-CAP participant hospitalized with CAP and interested in participation in the prospective “diseased” cohort is visited at home by a trained interviewer. Informed consent is obtained during this home visit. If consent is given, questions on health status, co-morbidities, current living situation and healthcare resources used before hospital admission are collected (see details in Table 2). A diary for own recording of healthcare resource use and other out-of-pocket expenses in the forthcoming period (i.e. one month) is explained and provided. Healthcare resources used during hospital admission (i.e. length of hospitalization, medication, diagnostics, interventions (e.g. surgery), complications, ICU admission) is collected from hospital databases by local trial nurses.

**Table 2:** **Data collection in matched cohort-study**^**a**^

**Cohort**	**Diseased cohort (i.e. CAP patients)** ***During home visit; Contact at baseline***	**Non-diseased cohort (i.e. unaffected (no CAP) persons)** ***During home visit; Contact at baseline***	**Diseased/non-diseased cohort** ***Per post; Contact at 1, 6 and 12 months***
**Data collected:**			
*Demographic data*	➣ Current living situation	➣ Current living situation	➣ Current living situation
*Quality-of-life data*			
➣ 5-item EQ-5D instrument & VAS [22]	➣ As on day of interview	As on day of interview	As on day of interview
	➣ Referring back to worst moment of CAP episode		
	➣ Referring back to time previous to CAP episode		
➣ 36-item SF-36D instrument [24,25]	n.a.^b^	➣ 36-item SF-36D instrument [24,25]	➣ 36-item SF-36D instrument[24,25]
*Co-morbidities*			
➣ Stroke and/or cardio-vascular event	➣ Since participating in CAPiTA;	➣ Since participating in CAPiTA,	➣ Since last contact moment;
➣ 28-item standard list of major comorbid diseases developed by Statistics Netherlands	➣ 28-item standard list of major comorbid diseases	➣ 28-item standard list of major comorbid diseases	n.a.^b^
*Medical resources used:*			
➣ Medication used	➣ Previous to hospital admission to treat CAP;	n.a.^b^	➣ Since last contact moment;
➣ GP; emergency department and other medical specialist consultations (phone; office and home consultation(s))	➣ Previous to hospital admission to treat CAP;	n.a.^b^	➣ Since last contact moment;
➣ Outpatient visit(s) and treatment(s)	n.a.^b^	n.a.^b^	➣ Since last contact moment;
➣ Hospital admission(s) and treatment(s)	n.a.^b^	n.a.^b^	➣ Since last contact moment;
➣ Institutional care admission(s) others than hospital	n.a.^b^	n.a.^b^	➣ Since last contact moment;
*Home care*			
➣ Professional home care used	➣ Previous to hospital admission during the CAP episode?	In month previous to interview	➣ Since last contact moment;
*Productivity losses due to absence from patient/control from unpaid work (including replacement)*			
➣ Is unpaid work conducted on a regular base.	➣ Previous to CAP hospital admission	n.a.^b^	➣ Since last contact moment;
➣ Absent from unpaid work: For how long. Who took the work over?	➣ Previous to hospital admission during the CAP episode?	n.a.^b^	➣ Since last contact moment;
*Productivity losses due to absence of caregiver(s) from (un)paid work*			
➣ Informal home care used: By who? And for how long?	➣ Previous to hospital admission during the CAP episode	n.a.^b^	Since last contact moment;
*Travel costs:*			
Method of transport and cost	➣ To hospital when admitted with CAP	n.a.^b^	n.a.^b^
	➣ After discharge from hospital to home/nursing home	n.a.^b^	n.a.^b^
*Other cost/out-of-pocket expenses* (open question)	n.a.^b^	n.a.^b^	➣ Since last contact moment;

Note:

a) Questionnaires (in Dutch) are available on request from first author.

b) n.a. = not applied.

Similar procedures are followed for the controls. An identified and eligible control who expressed interest to participate at the time of vaccination is visited at home. If consent is given, a questionnaire similar to that administered in the “diseased” cohort is provided (see details in Table 2).

In both the “diseased” and “non-diseased” cohorts, further data is collected on 1, 6 and 12 months after the initial home visit. Questionnaires are send by post, with the request to fill-in and send back within a week. If no response is received, participants are contacted by phone. This follow-up questionnaire addresses current living conditions, quality-of-life, co-morbidities and healthcare resources used (Table 2). A new diary for recording of healthcare use and other out-of-pocket expenses in the forthcoming period is provided once the questionnaire has been returned.

### Sample size

As CHO-CAP was designed and executed in parallel to the CAPiTA study, the sample size is - to a large extent – determined by the infrastructure of that study. The sample size of the different cohorts in CHO-CAP is determined by the expected number of CAP episodes occurring in the CAPiTA trial (i.e. ~ 2,000 cases, see [1]), as well as by the response rate obtained for the baseline CHO-CAP questionnaire and the willingness of patients with CAP and controls to participate in the nested matched cohort study. It is expected that at most 600 CAP episodes will be included in the “diseased” cohort and 1,200 controls in the “non-diseased” cohort.

### Data analysis

#### Descriptive statistics

The health status as observed in the baseline cohort and at different follow-up moments in the “diseased” and “non-diseased” cohorts will be presented, with and without stratification for age, gender and/or baseline EQ-5D scores. Following guidelines from the SF-36 developers [24,25], SF-36 results will be presented in a decomposed manner, i.e. for the 8 sub-scales and for the 2 composite summary scores for physical and mental functioning. We will calculate mean, median and the 5th, 25th, 75th and 95th percentiles for all QoL data.

The observed use of healthcare and other resources for CAP patients and controls over time will be described, in quantity (volumes of resource use) and in costs, following Dutch guidelines for health economic studies [26]. Costs will be presented, both undiscounted and discounted using different discount rates. Decomposed in volumes of resources use and associated costs, results will be presented as mean, median and the 5th, 25th, 75th and 95th percentile.

#### Statistical analysis

The main analysis will be the comparison of the matched cohort of CAP patients with the non-diseased controls. Univariate analysis will be applied in first instance. Depending on the distribution of data, we will use t-test or non-parametric tests (e.g. nonparametric bootstrap) to estimate the difference between both cohorts. Univariate analysis is also applied when analysing the health status for age groups within the baseline population. Here, ANOVA tests will be used as well. Where dealing with mainly normally distributed data, OLS (Ordinary least squares) regression is applied. Otherwise, the generalized linear model (GLM) will be preferred.

## Discussion

The infrastructure of the CAPiTA trial offers a unique opportunity for prospective collection of health outcomes, QoL and cost data of patients hospitalized with CAP. Such data will facilitate the construction and validation of health economic models on cost-effectiveness and cost-utility of 13vPnC in the future, should this become necessary if vaccine efficacy and safety is demonstrated.

One of the limitations of the current study is that we might mainly reach the ‘healthier’ part of the community-dwelling CAPiTA participants, as older and more diseased subjects may be less willing to participate in additional research projects next to the main CAPITA trial. To gain insight into this possible bias, we will compare the baseline demographic data of the CHO-CAP cohort population with the larger CAPiTA population. Further, data gathered within the CAPiTA-study will enable us to compare CAP cases from the current CHO-CAP cohort with all CAP cases, for instance with regard to duration of hospitalization and occurrence of complications.

With an expected 600 CAP episodes we expect to constitute one of the biggest prospectively studied cohorts of hospitalized elderly with CAP with regard to resources use and QoL data. Strengths of this study further include collection of out-of-pocket costs of patients and productivity losses of both patients and their caregivers and the follow-up period of up to one year post-discharge. Our study is expected to add more in-depth knowledge on the short and longer term outcomes of pneumonia in elderly.

## Endnote

^a^Commercial marketing questionnaires without any involvement of the participants normally reach a response rate around 20%. Healthcare questionnaires are known to obtain slightly higher response rates, whereby the greater the involvement, the higher the response rate. Given their previous participation in the CAPiTA study we expected some involvement from these participants for the current study, and therefore a response rate of 30% was expected to be feasible.

## Competing interests

The CHO-CAP study is made possible by an unrestricted grant from Wyeth Pharmaceuticals (now Pfizer Inc.) to the University Medical Center of Utrecht (UMCU). All three authors are employed by UMCU.

## Authors’ contributions

MJJM made substantial contributions to conception and design of the study, drafted the approved study protocol as well the current manuscript. MJMB was involved in the conception and design of the study and critically revised the current manuscript. GAdW made substantial contributions to conception and design of the study, has been involved in drafting the approved study protocol, and critically revised the current manuscript. All authors read and approved the final manuscript.

## Pre-publication history

The pre-publication history for this paper can be accessed here:

http://www.biomedcentral.com/1471-2334/13/597/prepub
